# Antimicrobial susceptibility patterns of clinical isolates of *Pseudomonas aeruginosa* isolated from patients of respiratory tract infections in a Tertiary Care Hospital, Peshawar

**DOI:** 10.12669/pjms.333.12416

**Published:** 2017

**Authors:** Abdul Samad, Tanveer Ahmed, Afaq Rahim, Abdul Khalil, Iftikhar Ali

**Affiliations:** 1 Prof. Abdul Samad, FRCP, Department of Medicine & Allied, Northwest General Hospital & Research Centre, Peshawar, Khyber Pakhtunkhwa, Pakistan; 2 Dr. Tanveer Ahmed, MBBS, Department of Medicine & Allied, Northwest General Hospital & Research Centre, Peshawar, Khyber Pakhtunkhwa, Pakistan; 3 Dr. Afaq Rahim, MBBS, Department of Medicine & Allied, Northwest General Hospital & Research Centre, Peshawar, Khyber Pakhtunkhwa, Pakistan; 4 Dr. Abdul Khalil, MCPS, Department of Medicine & Allied, Northwest General Hospital & Research Centre, Peshawar, Khyber Pakhtunkhwa, Pakistan; 5 Dr. Iftikhar Ali, PharmD, MPH. Department of Pharmacy Services, University of Swabi, Khyber Pakhtunkhwa, Pakistan. Northwest General Hospital & Research Centre, Peshawar, Khyber Pakhtunkhwa, Pakistan

**Keywords:** *Pseudomonas aeruginosa*, Antibiotic susceptibility, Sputum culture, Multi drug resistance, Clinical isolates

## Abstract

**Objective:**

This study aims to determine the prevalence and susceptibility pattern of *Pseudomonas aeruginosa* and multidrug-resistant (MDR) isolates in patients suffering from respiratory tract infection.

**Methods:**

A cross sectional study was conducted from January to December 2014 in Northwest General Hospital and Research Centre, Peshawar. A total of 615 sputum samples were collected from both in and out-patients. Sputum samples were collected as per standard procedure and were inoculated on Blood, MacConkey and Chocolate agar. The isolates were identified by standard protocols using biochemical tests. The antibiotic susceptibility pattern of each isolate was checked as per Clinical and Laboratory Standards Institute (CLSI) guidelines using *Kirby-Bauer’s* disc diffusion method.

**Results:**

Out of 615 sputum samples, 354 (57.56%) were culture positive. Out of these a total of 71 (20.05%) strains of *Pseudomonas* were isolated, where 54.93% was from males and 45.07% were from females (Mean age was 44.29 ± 22.72:). Highest sensitivity was seen to Amikacin (92.86%) followed by Meropenem (91.55%) while lowest sensitivity was seen to Cefoperazone + Sulbactam (16.9%). There were 39.44% MDR strains, out of which 25% were Extensively Drug Resistant (XDR) and 10.71% were Pan Drug Resistant (PDR). In vitro susceptibility of MDR isolates showed highest sensitivity to Amikacin (82.14%) followed by Carbapenems (78.57%). All MDR isolates were resistant to Cefoperazone + Sulbactam. Resistance to Piperacillin + Tazobactam was 96.43%.

**Conclusion:**

*Pseudomonas aeruginosa* is one of the commonly isolated organisms and it is becoming more resistant to commonly used antibiotics. Carbapenems and aminoglycosides were the two classes of drugs that showed highest activity against *Pseudomonas aeruginosa*.

## INTRODUCTION

Antibiotics when first introduced were considered as a miraculous drug. Unfortunately, most of the cheaper antibiotics lost their efficacy due to emergence of resistance among bacteria. Expensive and complicated antibiotics were introduced to tackle simple infections.^1^

*Pseudomonas aeruginosa* is an aerobic, non-fermenting, Gram-negative bacillus, which is most commonly involved in opportunistic nosocomial infections. *Pseudomonas aeruginosa* develops resistance against almost all antibiotics by several mechanisms like, multi-drug resistance efflux pumps, resistance genes, biofilm formation, aminoglycoside modifying enzymes and mutations in different chromosomal genes. Furthermore, exposures to broad spectrum antibiotics and patient to patient spread have added to the rapid increase in the isolation of resistant strains.^2^ Despite advances in health care and wide variety of antipseudomonal agents, life threatening infections caused by *Pseudomonas aeruginosa* are still considered as one of the major health problems. Emergence of infections caused by MDR and PDR strains increases morbidity, mortality and imposes an enormous burden on health care cost.^3^ The resistance pattern of bacteria changes over time and varies from place to place^4^; therefore, regular surveillance both nationally and locally is needed to treat the infection empirically and effectively.^5^

This study aimed to determine the prevalence of *Pseudomonas aeruginosa* in patients with lower respiratory tract infection, their antibiotic susceptibility pattern and frequency of MDR *Pseudomonas* isolates in a clinical setting of Northwest General Hospital and Research Centre Peshawar.

## METHODS

A cross sectional study, using consecutive sampling was conducted in Northwest General Hospital and Research Centre, Peshawar, from January to December 2014. Ethical approval for the study was obtained from the hospitals ethics committee. Total of 615 sputum samples were collected from both out and in-patients suffering from respiratory tract infections over 12 month period from January to December 2014. Both genders and all age groups were included in the study. After excluding respiratory tract commensals, non-available and duplicate reports, 354 samples were further analyzed. Culture reports that showed growth of *Pseudomonas aeruginosa* were included in this study.

Sputum samples were collected using sterile containers labelled with the patient’s medical registration number. These were then inoculated on Blood, MacConkey and Chocolate agar and incubated at 37°C for 24-48 hours. After obtaining growth, the organisms were identified by standard protocols using different identification and biochemical tests i.e. colony morphology Gram-staining, positive oxidase reaction, production of pyocyanin on Mueller-Hinton agar (Oxoid UK), citrate utilization and growth at 42°C.

Antibiotic susceptibility was checked by *Kirby-Bauer’s* disc diffusion method while sensitive and resistant organisms were marked after measuring zone of inhibition as per CLSI guidelines.^6^ The antibiotic discs that were used to identify the susceptibility pattern of the bacterial pathogens included: Meropenem (10 mcg), Amikacin (30 mcg), Ceftazidime (30 mcg), Cefoperazone/ Sulbactam combination (70 mcg), Ciprofloxacin (5 mcg), Gentamicin (10 mcg), Imipenem (10 mcg) and Piperacillin /Tazobactam (110 mcg). These antibiotics were used to categorize the microorganisms as susceptible, intermediate or resistant. Intermediate susceptibility was counted as susceptible in this study. Data including patients’ demographics (age, gender and nationality), microbial species (as recorded in the sputum culture reports) and the antibiotic susceptibility patterns of pathogens were collected. The results obtained were arranged and evaluated using Microsoft Excel 2013. These were expressed by descriptive statistics.

Multi Drug Resistance (MDR) was defined as non-susceptibility to at least one agent in three or more antimicrobial categories. Extensively Drug Resistant (XDR) was defined as non-susceptibility to at least one agent in all but two or fewer antimicrobial categories (i.e. bacterial isolates remain susceptible to only one or two categories) while Pan Drug Resistant (PDR) was defined as non-susceptibility to all agents in all antimicrobial categories tested.^7^

## RESULTS

During the study period, a total of 354 sputum cultures showed positive growth and a total of 71(20.05%) *Pseudomonas aeruginosa* strains were isolated, out of which 39 (54.93%) were from males and 32 (45.07%) were from females. The mean age was 44.29 ± 22.72 ranging from 8 months to 82 years, with most growths from 41-60 years of age (36.62%). Table-I depicts demographics characteristics of patients.

**Table-I:** Demographics characteristics of patients.

*Characteristics*	*n(%)*
Age (mean ± SD)	44.29 ± 22.72
***Age groups***	
0-20	10 (14.08%)
21-40	19 (26.76%)
41-60	26 (36.62%)
61-80	14 (19.72%)
80 and above	02 (2.82%)
***Gender***	
Male	39 (55%)
Female	32 (45%)
***Nationality***	
Pakistani	43 (60.56%)
Afghani	28 (39.44%)

**Fig.1:**
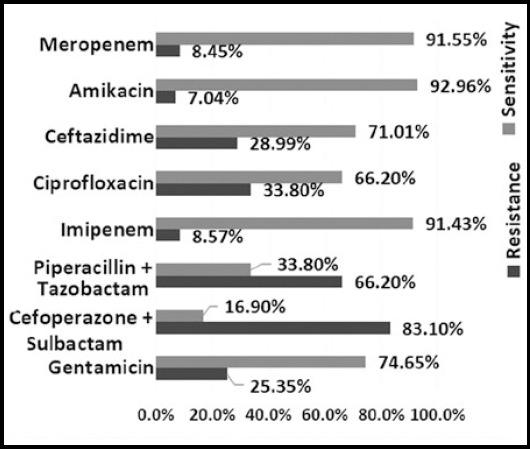
sensitivity and resistance pattern of *Pseudomonas aeruginosa*

*In vitro* antibiotic susceptibility of *Pseudomonas aeruginosa* showed highest sensitivity to Amikacin (92.86%) followed by Meropenem (91.55%) and Imipenem (91.43%) while showing lowest sensitivity to Cefoperazone + Sulbactam (16.90%). Sensitivity to Gentamicin was 74.65% and that of Ceftazidime was 71.01%. Highest resistance was seen to Cefoperazone + Sulbactam (83.10%) and Piperacillin + Tazobactam (66.20%). Overall sensitivity and Resistance pattern is shown in fig.1.

**Fig.2:**
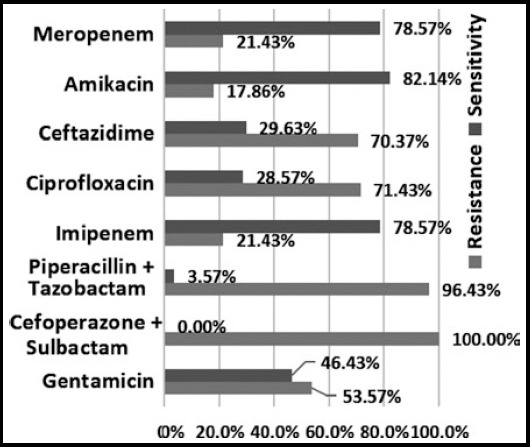
sensitivity and resistance pattern of *Pseudomonas aeruginosa* (mdr)

Out of 71 *Pseudomonas* isolates 28 (39.44%) were MDR, out of which 7 (25%) were XDR. PDR was seen in 3 (10.71%) of total MDR isolates. Highest sensitivity was seen to Amikacin (82.14%) followed by carbapenems (78.57%). All MDR isolates were resistant to cefoperazone + sulbactam. resistance to piperacillin + tazobactam was 96.43%. sensitivity and resistance pattern of MDR *Pseudomonas aeruginosa* is shown in Fig.2.

## DISCUSSION

Proper antibiotic administration is essential for the treatment of severe *Pseudomonas* infections. The resistance of *Pseudomonas* species to antibiotics has amplified considerably over the past few years and therefore need to be assessed regularly to have a clear opinion of clinical outcome of different therapeutic options.^8^

The result of the study revealed that *Pseudomonas* was seen in 20.05% of the total positive sputum cultures which is superior to a recent study done in Peshawar, Pakistan in 2015 by Abbas et al. Where they showed isolation from sputum samples to be 3.1%^9^ and inferior to a study done in North Waziristan, Pakistan in 2016 by Shah SN et al^10^ In a recent study done in Makkah, Saudi Arabia in 2016 by Ahmed et al. showed the frequency of *Pseudomonas aeruginosa* in sputum samples to be 38%,^11^ while another study done in Nepal in 2013 by Chander et al. showed 24.10%.^12^ In Iran by Anvari et al. in 2014 the isolation rate of *Pseudomonas* from sputum was 25%.^13^ Male predominance was seen in our study which is consistent with other studies published previously.^11^-^12^

This study also focused on the antibiotic susceptibility of *Pseudomonas* species. Aminoglycosides are broad spectrum antibiotics that act by inhibiting protein synthesis. These agents are used widely in various life threatening infections but have many side effects.^14^ In this study we found that the best drug against *Pseudomonas* is Amikacin. It showed 92.96% sensitivity, this is comparable to an international multicenter study done by Micek et al. In 2015^15^ while in contrast to Chander et al. 2013,^12^ Abbas et al. 2015^9^ and by Senthamarai et al. 2014.^3^ this may be due to selective use of aminoglycosides in our setup because of their higher adverse effects. Carbapenems are widely used against gram negative and gram positive microbes; sensitivity to Meropenem was 91.55% in our study which is better than a study done in India by Bajpai et al. in 2013^16^ and in Pakistan by Fatima et al. in 2012^17^ while other studies by Chander et al. in 2013^12^ in Nepal and by Sabir et al. in pakistan in 2014^18^ showed sensitivity of Carbapenems to be 100% against *Pseudomonas*, this shows that resistance is developing gradually, as it is being used now in several hospitals in our locality. Highest resistance was seen to Cefoperazone + Sulbactam (83.10%) in our study as opposed to Abbas et al. in 2015^9^ and Nadeem et al. In 2009.^19^ resistance to Piperacillin + Tazobactam was 66.20% which is also significantly higher than previously reported by Micek et al.2015,^15^ Abbas et al.2015^9^ and Fatima et al. 2012.^17^ Sensitivity to Ceftazidime and Ciprofloxacin was seen to be 71.01% and 66.20% respectively. In contrast, a study done in Punjab Pakistan by Sarwar et al. In 2013^8^ showed 22% sensitivity to Ceftazidime and 41.5% to Ciprofloxacin, while another study by Ahmed et al. In Saudi Arabia in 2016^11^ showed sensitivity to Ceftazidime and Ciprofloxacin to be 67.6% and 75.9% respectively, similar to our findings.

The frequency of MDR *Pseudomonas* in our study was 39.44% which is comparable to Abbas et al. In 2015.^9^ Another study done on MDR *Pseudomonas* in Rawalpindi, Pakistan by Gill et al. in 2010^2^ showed 19.5% MDR *Pseudomonas* isolated from sputum specimens. This shows an increase in resistance of *Pseudomonas* species with time. A study conducted in Karachi, Pakistan by Mansoor et al. in 2015 showed frequency of MDR *Pseudomonas* to be 36.59%.^20^ While another study done in Canada by Walkty et al. in 2013 showed isolation of MDR to be 6.5%.^21^ in *vitro* antibiotic susceptibility of MDR *Pseudomonas* showed highest sensitivity to Amikacin 82.14% which is similar to gill et al., 2010.^2^ The MDR isolates showed 100% and 96.43% resistance to Cefoperazone + Sulbactam and Piperacillin + Tazobactam respectively, which is antithetical to Mansoor et al. in 2015^20^ where the aforementioned drugs had better sensitivities.

### Limitations

The study showed the frequency of *Pseudomonas* with their antibiotic susceptibilities. There were certain limitations in our study that could not be avoided. The data included in this study was only from a single hospital and thus does not reflect the whole population of our area. Therefore, a survey of a larger sample size from different centers would give us a more accurate representation of our population. There was no segregation between community acquired and nosocomial infections. Furthermore, our specimens were only limited to sputum isolates. Co-morbidities were not considered, which would give an insight on the relationship with *Pseudomonas* infection. It is also possible that MDR *Pseudomonas* can be attributed to the patients in intensive care units. A breakdown of patient distribution throughout the hospital was not taken into account in this study.

## CONCLUSION

this study concluded that *Pseudomonas aeruginosa* is one of the commonly isolated organisms and it is becoming more resistant to commonly used antibiotics. Carbapenems and aminoglycosides were the two classes of drugs that showed best activity against *Pseudomonas*. The frequency of MDR strains in *Pseudomonas* is also on the rise. Since this study is limited to only one center in Peshawar, it is recommended to conduct a large scale study to find out the exact resistance pattern of our population. More rational use of antibiotics is required to counter the developing resistance among bacteria.
